# Pamphlets Received

**Published:** 1876-09

**Authors:** 


PAMPHLETS RECEIVED.—A sketch of Louyse Bourgeois, Midwife to Marie De'Medici, the Queen of Henry the IV. of France. The Annual Address of the retiring President before the Philadelphia County Medical Society. By William Goodell A.M., M.D. Delivered June 5, 1876.

This is quite an interesting paper, showing the many quaint ideas entertained by midwives of that time. The paper has many quotations from the writings of the eccentric, hardsensed, persevering Louyse, giving the incidents which led to her admission as midwife to the Queen, which are quite laughable, but throughout show her wonderful good sense and policy.

Anchylosis of the tempro maxillary articulation of long standing; fracture of the right condyle; atrophy of the depressor and contraction of the elevator muscle of the inferior maxillary. By D. H. Goodwillie, D.D., M.D., Clinical Assistant to the Metropolitan Throat Hospital, Permanent Member of American Medical Association, etc. (Reprint.)

Thirty-seven operations of Thoracentesis, by Pneumatic Aspiration. By Frank Donaldson, M.D., Clinical Professor of Diseases of the Throat and Chest, University of Maryland. (Reprint.) The thirty-seven operations were upon twenty-two patients. Two deaths in tuberculous cases. Five of the cases had empyemia from chronic pleurisy; in seventeen the effusions were serous in character. He thinks the operation not very dangerous, should be resorted to where the effusions are not removed by other means, and should not wait too long; in acute cases operates where danger is imminent from rapid effusions. No doubt the operation will come into more general use.

An Address on some of the leading Public Health Questions; with remarks on the extent of swamp lands in the United States, and their reclamation as a sanitary and economic measure, delivered at the opening of the third annual meeting of the American Public Health Association, at Baltimore, Nov. 9, 1875. By J. M. Toner, M.D., President of the Association. (Reprint.)

Analysis of Six Hundred and Seventeen Cases of Skin Disease, with cases and remarks on treatment. Being a study of the cases of Diseases of the skin treated at Demlit Dispensary during the year 1875. By L. Duncan Bulkly, A.M., M.D., Physician to the Skin Department, Demlit Dispensary, New York, etc. (Reprint.)

Orthopedic Surgery; Deformities of the Lower Extremities. By Van S. Lindsley, M.D., Professor of Physiology in University of Nashville and Vanderbilt University. Read before Medical Society of Tennessee, April, 1876.

Remarks on Intra-Uterine Polypi, with special reference to their diagnosis and surgical treatment. By A. Reeves Jackson, A.M., M.D., Surgeon-in-Chief of the Woman's Hospital of the State of Illinois, etc. (Reprint.)

The Climatotherapy of, and the American Mountain Sanitariam for, Consumption. By Stanford E. Chaille, A.M., M.D., Professor of Physiology and Pathological Anatomy Medical Department University of Louisiana. (Reprint.)

Western North Carolina as a Health Resort. By W. Gleitsman, M.D., Physician in Charge of the Mountain Sanitarium for Pulmonary Diseases, Ashville, N. C. Read before the American Public Health Association at Baltimore. (Reprint.)

Michigan State Medical Society. Address of the President, Dr. William Brodie, of Detroit. Delivered at Ann Harbor, May 10, 1876. (Reprint.)

International Medical Congress, Philadelphia, September 4–9, 1876. Outlines of Papers Presented by the Reporter on Questions Assigned for Discussion in the Sections.

